# Perfusion Patterns of Peripheral Pulmonary Metastasis Using Contrast-Enhanced Ultrasound (CEUS) and Their Correlation with Immunohistochemically Detected Vascularization Pattern

**DOI:** 10.3390/cancers16193365

**Published:** 2024-10-01

**Authors:** Johannes Kroenig, Christian Görg, Helmut Prosch, Lara Von Schumann, Christina C. Westhoff, Amjad Alhyari, Felix R. M. Koenig, Hajo Findeisen, Ehsan Safai Zadeh

**Affiliations:** 1 Lung Center Mainz, Clinic for Pneumology, Center for Thoracic Diseases, University Medical Center Mainz, 55131 Mainz, Germany; johannes.kroenig@unimedizin-mainz.de; 2 Interdisciplinary Center of Ultrasound Diagnostics, Gastroenterology, Endocrinology, Metabolism and Clinical Infectiology, University Hospital Giessen and Marburg, Philipp University of Marburg, Baldingerstraße, 35033 Marburg, Germanyalhyari@med.uni-marburg.de (A.A.); 3 Department of Biomedical Imaging and Image-Guided Therapy, Medical University of Vienna, 1090 Wien, Austria; helmut.prosch@meduniwien.ac.at (H.P.); felix.koenig@meduniwien.ac.at (F.R.M.K.); 4 Institute of Pathology, University Hospital Giessen and Marburg, Philipps University Marburg, Baldingerstraße, 35043 Marburg, Germany; 5 Department for Internal Medicine, Red Cross Hospital Bremen, 28209 Bremen, Germany

**Keywords:** peripheral pulmonary metastasis, contrast-enhanced ultrasound, lung ultrasound, vascularization

## Abstract

**Simple Summary:**

This study investigated how pulmonary metastases in the lungs can be assessed using a special imaging technique called contrast-enhanced ultrasound (CEUS). We analyzed data from 54 patients with confirmed cancer spread to the lungs. Our goal was to identify patterns of blood flow in the lung tumors and to compare these with the findings from tissue samples stained to highlight blood vessels. The results showed that most lung tumors had a blood supply from the bronchial arteries, with a few showing a supply from the pulmonary arteries. Additionally, most tumors showed a rapid reduction in contrast enhancement. This research helps improve the understanding of blood flow in lung tumors and could aid in developing better diagnostics strategies.

**Abstract:**

Purpose: Description of the perfusion of pulmonary metastasis by contrast-enhanced ultrasound (CEUS) and their correlation with vascularization patterns represented by immunohistochemical CD34 endothelial staining. Patients and methods: The data of 54 patients with histologic proven peripheral pulmonary metastasis, investigated between 2004 and 2023 by CEUS. These CEUS parameters were evaluated: time to enhancement (TE), categorized as early pulmonary-arterial (PA) or delayed bronchial-arterial (BA) patterns; extent of enhancement (EE), either marked or reduced; homogeneity of enhancement (HE), homogeneous or inhomogeneous; and decrease of enhancement (DE), rapid washout (<120 s) or late washout (≥120 s). Additionally, tissue samples in 45 cases (83.3%) were stained with CD34 antibody for immunohistochemical analysis. Results: In total, 4 lesions (7.4 %) exhibited PA enhancement, and 50 lesions (92.6%) demonstrated BA enhancement. Furthermore, 37 lesions (68.5%) showed marked enhancement, while 17 lesions (31.5%) exhibited reduced enhancement. The enhancement was homogeneous in 28 lesions (51.86%) and inhomogeneous in 26 lesions (48.14%). Additionally, 53 lesions (98.1%) displayed a rapid washout. A chaotic vascular pattern indicative of a bronchial arterial blood supply was identified in all cases (45/45, 100%), including all 4 lesions with PA enhancement. Conclusion: Pulmonary metastases in CEUS predominantly reveal bronchial arterial enhancement and a rapid washout. Regarding EE and HE, pulmonary metastases show heterogeneous perfusion patterns. A PA enhancement in CEUS does not exclude BA neoangiogenesis.

## 1. Introduction

Lung metastases are relatively prevalent in patients with underlying malignant diseases. The literature reports an incidence rate of 18 per 100,000 for synchronous lung metastases [1]. Therefore, the evaluation of malignancy in pulmonary masses in these patients holds significant diagnostic and prognostic importance. Computed tomography (CT) and positron emission tomography–computed tomography (PET-CT) are the imaging modalities of choice for assessing indeterminate pulmonary nodules [2]. CT and PET-CT exhibit high sensitivities of 94% and 89%, respectively, but moderate specificities of 73% and 78% in differentiating benign from malignant solitary pulmonary nodules [3]. Consequently, the advancement of diagnostic strategies to further characterize indeterminate pulmonary masses remains imperative. Lung ultrasound (LUS) presents several limitations to the evaluation of pulmonary lesions. It is restricted to visualizing pleura-adjacent lesions, and due to interference from air and bony structures, only approximately 70% of the pleural surface is accessible via LUS [4]. Nevertheless, if a peripheral pulmonary lesion is detectable on LUS, particularly with contrast-enhanced ultrasound (CEUS), this modality can provide valuable supplementary information to cross-sectional imaging [5,6,7,8,9,10,11,12,13,14,15,16,17,18,19,20,21,22,23,24]. Ultrasound has the highest spatial resolution compared to other imaging modalities, and the contrast agent in CEUS remains strictly intravascular [25,26,27,28,29]. These features allow for the dynamic visualization of the perfusion of lesions. Preliminary studies indicate that CEUS may be beneficial for distinguishing between acute and chronic processes [30,31]. To explore the clinical importance and potentially integrate CEUS into clinical practice, it is essential to first investigate and delineate the foundational principles of CEUS across various subgroups. Currently, there are limited data regarding the CEUS patterns of lung metastases. This study aims to elucidate the fundamentals of CEUS in the subgroup of non-hematologic pulmonary metastases, correlating with histopathological findings.

## 2. Materials and Methods

Between 2004 and 2023, a total of 69 patients with suspected pleura-based non-hematological pulmonary metastases (PM), visualizable by B-mode lung ultrasound (B-LUS), were analyzed using contrast-enhanced ultrasound (CEUS). The patients presented to the ultrasound center either for the histological confirmation of peripheral pulmonary lesions or for correlation with cross-sectional imaging. The examinations were standardized and conducted by a single examiner, qualified at DEGUM Level III from the German Society for Ultrasound in Medicine, with over 35 years of experience in thoracic sonography (C.G., internal medicine) at a university ultrasound center. All pleural-based lesions (PPLs) were larger than 5 mm. The inclusion criteria for this retrospective analysis were (1) histological confirmation of lesions as non-hematological pulmonary metastases and (2) standardized documentation of the B-LUS and CEUS data. A total of 15 patients were excluded because they did not meet the study criteria of histologically confirmed pulmonary metastasis (n = 2 no histological confirmation, n = 10 primary tumor was identified). Ultimately, 54 patients with proven non-hematological metastases met the inclusion criteria and were included in the study. Informed consent for the CEUS examination was obtained from all patients, and the study received approval from the local ethics committee (protocol code: 24-204 RS), being conducted in accordance with the revised Declaration of Helsinki.

### 2.1. Ultrasound

B-LUS examinations were carried out using an ACUSON SEQUOIA 512 GI ultrasound machine (Siemens, Erlangen, Germany) with a 4C1 curved-array transducer at 4 MHz. CEUS was performed with the same transducer in contrast-specific mode (1.5 MHz), following EFSUMB guidelines [32]. A 2.4 mL bolus of SonoVue^®^ (Bracco Imaging S.p.A., Milan, Italy) was administered intravenously, followed by 10 mL of NaCl 0.9%. Lesion perfusion was continuously monitored for 30 s, recorded as clips, and then, re-evaluated at one-minute intervals for up to 3 min, with changes documented as images. All examinations were conducted in a sitting position, parallel to the ribs. The following B-LUS and CEUS parameters were analyzed retrospectively.

### 2.2. B-Mode Ultrasound Parameter

1. The echogenicity of the lesion was classified as either hypoechoic or iso-/hyperechoic in comparison to the echogenicity of parenchymal organs, which served as an in vivo reference;
2. The size of the peripheral pulmonary lesion was measured in centimeters.

### 2.3. Contrast-Enhanced Ultrasound Parameters

The parameters collected to determine the perfusion pattern using contrast media were as follows.

1. The time to enhancement (TE) following intravenous contrast injection was measured and categorized into two patterns, namely early pulmonary-arterial (PA) enhancement, where the lesion showed contrast enhancement before it appeared in the thoracic wall, and delayed bronchial-arterial (BA) enhancement, where contrast enhancement appeared in the lesion at the same time or after it reached the thoracic wall or parenchymal organs [33];
2. The extent of enhancement (EE) during the arterial phase was classified as either reduced EE (hypoechoic) or marked EE (isoechoic), compared to the parenchymal enhancement of the spleen, which was used as an in vivo reference [33];
3. The homogeneity of enhancement (HE) during the arterial phase was categorized as either homogeneous or inhomogeneous. Lesions showing both perfused and non-perfused areas (NPAs) were classified as having inhomogeneous enhancement [33];
4. The decrease of enhancement (DE) in the parenchymal phase (washout) was classified as either a rapid washout (<120 s) or a late washout (>120 s) [34].

The B-LUS and CEUS data were retrospectively evaluated by two independent, experienced investigators (E.S. and C.G.). In the event of discrepancies, the final decision was made by a third experienced investigator (H.F.).

### 2.4. Histopathological Examinations

**Figure 1:**
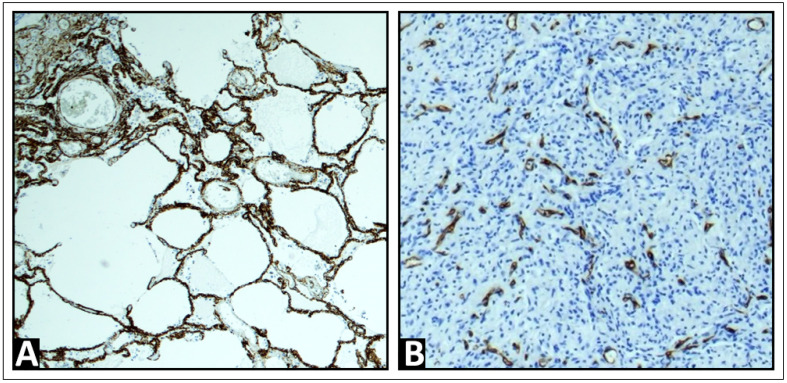
Exemplary immunohistochemistry patterns for CD34, a marker of endothelial cells, were observed. (**A**) A regular alveolar pattern, corresponding to the pulmonary capillary network in healthy lung tissue, is indicative of pulmonary arterial supply. (**B**) A disorganized and chaotic vascular pattern, characteristic of bronchial arterial neoangiogenesis, is indicative of bronchial arterial supply.

In all cases, tissue samples were obtained through either US-guided biopsy or surgical sampling. The samples were fixed in a 4% formalin solution, embedded in paraffin, sectioned at a thickness of 4 μm, and stained with hematoxylin and eosin (H&E) for routine analysis. In 45 of the 54 cases, immunohistochemistry for CD34, an endothelial cell marker, was performed to assist in the evaluation of the vascular architecture and to confirm the presence of malignant cells [35,36,37,38] using standard methods (EnVision + Dual Link System-HRP, with 3,3′-diaminobenzidine as chromogen) and detected by the monoclonal antibody QBEnd10 (Agilent Dako, Waldbronn, Germany). An experienced pathologist (C.C.W.) microscopically identified all tissue samples as diseased lung tissue with metastases. The vascular patterns observed included a regular alveolar pattern, consistent with the pulmonary capillary network found in healthy lung tissue or acute pneumonia, indicating PA supply (Figure 1A). In contrast, disorganized and chaotic vascular patterns, resembling the BA neo-angiogenesis typical of malignant lung tumors, were identified for BA supply, as previously described (Figure 1B) [39].

## 3. Results

Of 54 patients, 29 were males and 25 were females. The mean age was 68.74 years, with a standard deviation of 12.8 years (range 29–88 years). The tumor entities confirmed in these patients were non-small cell lung cancer (NSCLC) (n = 12), colorectal cancer (CRC) (n = 7), renal cell carcinoma (RCC) (n = 6), breast cancer (n = 4), pancreatic cancer (n = 3), sarcoma (n = 3), head/neck carcinoma (n = 7), and gynecological carcinoma (cervical, endometrial, vulvar, and uterine) (n = 6), germ cell tumor (n = 2) as well as individual cases of cancer of unknown primary (CUP), melanoma, hepatocellular carcinoma (HCC), and urothelial carcinoma(Table 1). Histologically, there were 50 carcinomas, 3 sarcomas, and 1 melanoma. A total of 53 lesions were confirmed by biopsy, and one case was confirmed surgically.

### 3.1. B-Mode Ultrasound Data

All 54 lesions appeared hypoechoic on B-LUS. The average size of the lesions was 2.0 × 1.9 cm, with a range from 0.4 × 0.4 cm to 5 × 4 cm.

### 3.2. Contrast-Enhanced Ultrasound Data

**Figure 2:**
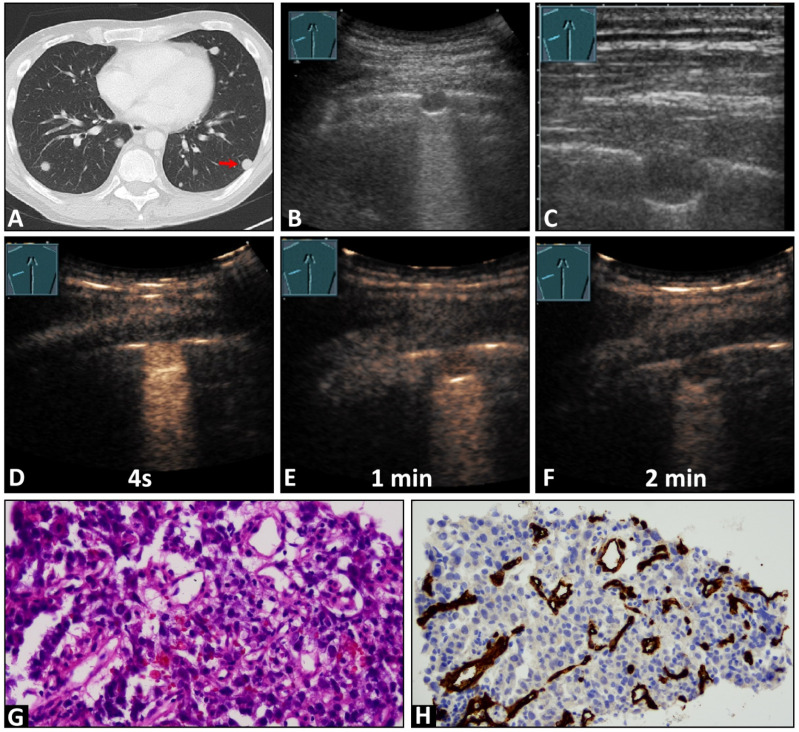
A 45-year-old male patient with a known previously diagnosed non-seminomatous germ cell tumor and multiple pulmonary nodules on (**A**) computed tomography (arrow) and (**B**,**C**) B-mode ultrasound. An ultrasound-guided 18 G core needle biopsy of the lung lesion was performed, and histopathological examination confirmed the diagnosis of a metastatic germ cell tumor. On contrast-enhanced ultrasound, the lesion displayed (**D**) early enhancement, indicative of a pulmonary-arterial supply, and (**D**) a marked and homogeneous pattern of enhancement with (**E**,**F**) an early decrease of enhancement. (**G**) The tissue sample (HE staining) showed infiltration by a malignant, epithelial differentiated tumor. (**H**) Immunohistochemical staining with CD34 was performed, revealing a chaotic pattern, consistent with bronchial arterial neoangiogenesis (×200).

**Figure 3:**
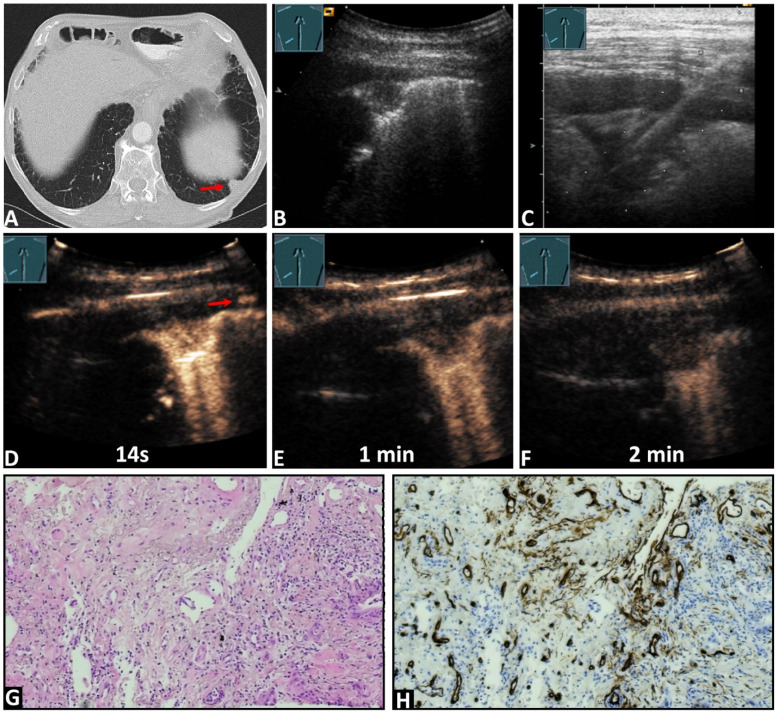
An 84-year-old male patient with known pancreatic carcinoma and a pulmonary nodule on the (**A**) computed tomography (arrow) (courtesy of Prof. Dr. Andreas H. Mahnken, Department of Radiology, University Hospital Marburg) and (**B**,**C**) B-mode ultrasound. (**C**) An ultrasound-guided 18G core needle biopsy of the lung lesion was performed, and histopathological examination confirmed the diagnosis of metastatic pancreatic carcinoma. On contrast-enhanced ultrasound, the lesion demonstrated (**D**) an enhancement simultaneous with the arrival of the contrast agent in the intercostal artery (arrows), and a marked and homogeneous pattern of enhancement with (**E**,**F**) an early decrease of enhancement. (**G**) The tissue sample (HE staining) showed the infiltrates of a ductal adenocarcinoma consistent with a metastasis of the previously known pancreatic carcinoma. (**H**) immunohistochemical staining with CD34 was performed, revealing a chaotic pattern consistent with bronchial-arterial neoangiogenesis (×100).

In total, 4 lesions (7.4%) exhibited PA enhancement (Figure 2), and 50 lesions (92.6%) demonstrated BA enhancement (Figure 3). Furthermore, 37 lesions (68.5%) showed marked enhancement, while 17 lesions (31.5%) exhibited reduced enhancement. The enhancement was homogeneous in 28 lesions (51.86%) and inhomogeneous in 26 lesions (48.14%). Additionally, 53 lesions (98.1%) displayed a rapid washout, and one lesion (1.9%) (RCC) exhibited a late washout.

### 3.3. Histopathological Data and Their Correlation with Contrast-Enhanced Ultrasound Pattern

In 45 cases (83.3%), immunohistochemical staining with CD34 endothelial staining was performed. A chaotic vascular pattern indicative of a bronchial arterial blood supply was identified in all cases (45/45, 100%, Figure 2 and Figure 3). In 7/45 (15.5%) samples, an organized vascularization along the alveoli, consistent with a pulmonary arterial supply pattern, was also observed. In four cases with a PA perfusion pattern of enhancement in CEUS, 2/4 lesions (50%) exhibited both organized PA vascularization and chaotic BA vascularization, while in the remaining 2/4 lesions (50%), only chaotic BA vascularization was observed (Figure 3). Table 2 presents the histopathological findings with the corresponding CEUS patterns.

## 4. Discussion

The lung has a dual blood supply [40,41,42,43]. The pulmonary arterial supply originates from the right ventricle and extends to the alveolar capillaries, where oxygenation occurs. The bronchial arteries, originating from the aorta, perfuse the entire lung parenchyma (vasa privata) [44,45]. The nutritive supply of a lung lesion can be provided by either the right (pulmonary arterial) or left (bronchial arterial) heart system [33,44,45]. In healthy lung tissue or acute inflammatory processes, where the lung architecture is still intact, the pulmonary arterial supply is the dominant vascularization [30,34,46]. In contrast, in chronic processes, such as bronchial carcinomas, granulomatous lesions, and organized pneumonias, the lung architecture is destroyed, and neoangiogenesis occurs through bronchial arterial branches, making the bronchial artery the dominant vascularization [39,44,45,47,48]. Basic studies show that a distinction can be made between the pulmonary arterial supply and the bronchial arterial supply of a peripheral lung lesion in CEUS [30,34,39,46]. In the case of a pulmonary arterial supply, the strictly intravascular contrast agent in CEUS reaches the pulmonary lesions before all other organs with systemic vascularization through the left ventricle (e.g., thoracic wall, spleen, liver) [33]. In the case of a bronchial arterial supply, the contrast agent in CEUS reaches the pulmonary lesions simultaneously with all other organs, with systemic vascularization through the left ventricle (e.g., thoracic wall, spleen, and liver) [33].

Pulmonary metastases, as lesions with neoangiogenesis, predominantly showed bronchial arterial enhancement in 92.6% of cases. In all lesions with immunohistochemical endothelial CD34 staining, a chaotic vascularization, consistent with bronchial arterial vascularization, was detected. Among the four lesions with PA enhancement, two cases showed a mixed pattern of chaotic BA supply and organized PA supply in CD34 staining, while the other two cases showed only a chaotic BA arterial supply. The reason for this discrepancy (bronchial arterial pattern in immunohistochemical CD34 staining and PA arterial enhancement in CEUS) might be the presence of tiny shunts between the pulmonary arterial and bronchial arterial vessels that may not be visible in small needle biopsy samples. It is well known that numerous pre- and post-capillary anastomoses develop, particularly under hypoxic conditions [49]. A PA enhancement can mask a BA enhancement on CEUS because the PA perfusion occurs earlier than the BA perfusion. These findings are important, as they demonstrate one of the limitations of contrast-enhanced ultrasound in the detection of chronic processes and show that PA arterial enhancement does not exclude BA neoangiogenesis as the vascularization pattern of pulmonary metastases.

Regarding the EE and HE, the lesions exhibited a heterogeneous pattern of perfusion. In 51.9% of cases, homogeneous enhancement was observed, while in 48.1%, inhomogeneous enhancement was noted. Furthermore, 68.5% showed marked EE, while 31.5% exhibited reduced enhancement. Inhomogeneous enhancement correlates to avascular areas within the tumor, and EE indicates the strength of vascularization of the lesions. These features vary depending on the tumor size and type, differing among various tumors [50].

Regarding the DE, 98.1% of lesions showed a rapid decrease in enhancement (<120 s). These findings are consistent with previous studies. Caremani et al. demonstrated that the presence of late DE is a sign of benign pulmonary lesions [34]. This may be due to the abnormal vessels and arteriovenous shunts that can be seen in the vascular networks generated by neoangiogenesis [51]. Only one renal cell carcinoma (RCC) metastasis showed a late washout. RCC is a highly vascularized malignant tumor characterized by extensive angiogenesis. The reason for the delayed washout may be the retention of the contrast agent within the lesion due to its extensive vascularization, which can mask the washout [52].

The results of this study indicate that pulmonary metastases predominantly show bronchial artery enhancement with rapid washout, and the perfusion of these lesions is consistent with primary bronchial carcinomas and other chronic processes, such as granulomatous lesions and organized pneumonia (Table 3). However, it demonstrates that the behavior of pulmonary metastases is in discrepancy with pulmonary hematologic lesions, acute pneumonia, or atelectasis, which predominantly show pulmonary artery perfusion with delayed washout. Table 3 presents the perfusion patterns of pulmonary metastases compared to other pulmonary lesions in CEUS.

The findings of this study could be significant in specific clinical situations, for example, for differentiating between acute pneumonia and metastasis in the appropriate clinical context. Another important point is that a pulmonary artery perfusion does not exclude pulmonary metastasis, and this limitation of CEUS must always be considered. However, a bronchial artery perfusion with rapid washout in lung lesions should be seen as an indication of pulmonary metastasis in patients with an underlying malignant disease. In the absence of an underlying malignant disease, bronchial arterial perfusion is an indication of the presence of a chronic process.

There are some limitations to mention, apart from the retrospective nature of the study. Ten patients in the presented cohort had pulmonary metastasized non-small cell lung carcinoma. Ultimately, it cannot be ruled out with absolute certainty that the initial primary and not the synchronous metastasis was punctured. Furthermore, a certain differentiation between a metastasis and a secondary lung cancer is not possible. The same applies to some squamous cell carcinomas. Naturally, a primary secondary carcinoma can always be present in these cases. Thoracic ultrasound is subject to natural limitations, meaning that it cannot image the entire surface of the lungs and requires a high level of examination expertise. Consequently, interobserver and interequipment variability must be assumed.

## 5. Conclusions

Pulmonary metastases in CEUS predominantly reveal bronchial arterial enhancement and a rapid washout. Regarding EE and HE, pulmonary metastases show heterogeneous perfusion patterns. These findings are consistent with primary bronchial carcinomas but are in discrepancy with hematologic lung lesions such as pulmonary lymphoma. These results show that, even within neoplastic lesions, different perfusion patterns can be observed among various subgroups. Furthermore, histopathological correlation demonstrated that PA enhancement does not exclude BA neoangiogenesis in pulmonary metastases. This limitation of CEUS in evaluating lung lesions must always be taken into account.

## Figures and Tables

**Table 1:** Histology of n = 54 patients.

Histology	Patients
carcinoma	50
sarcoma	3
melanoma	1

**Table 2:** Histopathological correlation with CEUS patterns.

No. of Cases	Vascular Pattern in CD34	Corresponding Perfusion Pattern on CEUS
**41**	BA vascular pattern	BA Perfusion
**2**	BA vascular pattern	PA Perfusion
**2**	BA and PA vascular pattern	PA Perfusion

BA: bronchial arterial; CEUS: contrast-enhanced ultrasound; PA: pulmonary arterial.

**Table 3:** CEUS perfusion patterns of pulmonary metastases in contrast to other lung pathologies.

Underlying Disease	Pulmonary Metastases	Central Lung Cancer	Peripheral Lung Cancer	Lung Lymphoma	Obstructive Atelectasis	Acute Pneumonia	Granulomatous Disease	Organized Pneumonia
Author	Present study	Safai Zadeh et al. [53]	Findeisen et al. [54]	Trenker et al. [55]	Safai Zadeh et al. [53]	Linde et al. [31]	Safai Zadeh et al. [39]	Safai Zadeh et al. [39]
No. of cases	54	48	89	6	54	50	10	38
Year	2024	2024	2019	2018	2024	2012	2021	2021
	Pattern of enhancement on CEUS							
TE: PA BA	7.4% 92.6%	10.4% 89.6%	28.1% 71.9%	83.3% 16.7%	85.2% 14.8%	92.0% 8.0%	0% 100%	28.9% 71.1%
EE: Marked Reduced	68.5% 31.5%	8.3% 91.7%	59.5% 40.5%	100% 0.0%	n.a.	74.0% 26.0%	0% 100%	76.3% 23.7%
HE: Hom Inhom	51.86% 48.14%	91.7% 8.3%	23.6% 76.4%	66.7% 33.3%	72.2% 27.8%	78.0% 22.0%	0% 100%	18.4% 81.6%
DE: Rapid Late	98.1% 1.9%	79.2% 20.8%	n.a.	50.0% 50.0%	27.8% 72.2%	n.a.	100% 0%	50.0% 50.0%

BA: bronchial arterial; CEUS: contrast-enhanced ultrasound; DE: decrease in enhancement; EE: extent of enhancement; HE: homogeneity of enhancement; Hom: homogeneous; Inhom: inhomogeneous; n.a.: not analyzed; PA: pulmonary arterial; OE: order of enhancement; TE: time to enhancement.

## Data Availability

The data presented in this study are available in this article.

## References

[1] Chen H., Stoltzfus K.C., Lehrer E.J., Horn S.R., Siva S., Trifiletti D.M., Meng M.B., Verma V., Louie A.V., Zaorsky N.G. (2021). The Epidemiology of Lung Metastases. Front. Med..

[2] McNulty W., Baldwin D. (2019). Management of pulmonary nodules. BJR Open.

[3] Jia Y., Gong W., Zhang Z., Tu G., Li J., Xiong F., Hou H., Zhang Y., Wu M., Zhang L. (2019). Comparing the diagnostic value of (18)F-FDG-PET/CT versus CT for differentiating benign and malignant solitary pulmonary nodules: A meta-analysis. J. Thorac. Dis..

[4] Messina G., Bove M., Natale G., Di Filippo V., Opromolla G., Rainone A., Leonardi B., Martone M., Fiorelli A., Vicidomini G. (2023). Diagnosis of malignant pleural disease: Ultrasound as “a detective probe”. Thorac. Cancer.

[5] Jung E.M., Stroszczynski C., Jung F. (2020). Contrast enhanced ultrasound (CEUS) to assess pleural pulmonal changes in severe COVID-19 infection: First results. Clin. Hemorheol. Microcirc..

[6] Li Q., Nie F., Yang D., Dong T., Liu T., Wang Y. (2022). Role of Contrast-Enhanced Ultrasound in Pulmonary Lesions: 5-Year Experience at a Single Center. Ultrasound Med. Biol..

[7] Wang S., Yang W., Zhang H., Xu Q., Yan K. (2015). The Role of Contrast-Enhanced Ultrasound in Selection Indication and Improveing Diagnosis for Transthoracic Biopsy in Peripheral Pulmonary and Mediastinal Lesions. BioMed Res. Int..

[8] Sperandeo M., Sperandeo G., Varriale A., Filabozzi P., Decuzzi M., Dimitri L., Vendemiale G. (2006). Contrast-enhanced ultrasound (CEUS) for the study of peripheral lung lesions: A preliminary study. Ultrasound Med. Biol..

[9] Boccatonda A., Andreetto L., Vicari S., Campello E., Simioni P., Ageno W. (2024). The Diagnostic Role of Lung Ultrasound and Contrast-Enhanced Ultrasound in Pulmonary Embolism. Semin. Thromb. Hemost..

[10] Liang J., Wang D., Li H., Zhao S., Chen M., Li H., Ding Z., Liu J., Liu L. (2020). Contrast-enhanced ultrasound for needle biopsy of thoracic lesions. Oncol. Lett..

[11] Bai Z., Liu T., Liu W., Li Z., Zheng H., Li X. (2022). Application value of contrast-enhanced ultrasound in the diagnosis of peripheral pulmonary focal lesions. Medicine.

[12] Wang Y., Xu Z., Huang H., Zhou X., Xian M. (2020). Application of quantitative contrast-enhanced ultrasound for evaluation and guiding biopsy of peripheral pulmonary lesions: A preliminary study. Clin. Radiol..

[13] Bai J., Du Y.Q., Yang W., Bai X.M., Wang S., Wu W., Yan K., Chen M.H. (2023). The Role of Contrast-Enhanced Ultrasound Plus Color Parametric Imaging in the Differential Diagnosis of Subpleural Pulmonary Lesions. J. Ultrasound Med..

[14] Bi K., Zhou R.R., Zhang Y., Shen M.J., Chen H.W., Cong Y., Zhu H.M., Tang C.H., Yuan J., Wang Y. (2021). US Contrast Agent Arrival Time Difference Ratio for Benign versus Malignant Subpleural Pulmonary Lesions. Radiology.

[15] Boccatonda A., Guagnano M.T., D’Ardes D., Cipollone F., Vetrugno L., Schiavone C., Piscaglia F., Serra C. (2024). The Role of Contrast-Enhanced Ultrasound in the Differential Diagnosis of Malignant and Benign Subpleural Lung Lesions. J. Clin. Med..

[16] Jiménez-Serrano S., Páez-Carpio A., Doménech-Ximenos B., Cornellas L., Sánchez M., Revzin M.V., Vollmer I. (2024). Conventional and Contrast-enhanced US of the Lung: From Performance to Diagnosis. Radiographics.

[17] Yusuf G.T., Fang C., Tran S., Rao D., Bartlett-Pestell S., Stefanidis K., Huang D.Y., Sidhu P.S. (2021). A pictorial review of the utility of CEUS in thoracic biopsies. Insights Into Imaging.

[18] Tang M., Xie Q., Wang J., Zhai X., Lin H., Zheng X., Wei G., Tang Y., Zeng F., Chu Y. (2020). Time Difference of Arrival on Contrast-Enhanced Ultrasound in Distinguishing Benign Inflammation from Malignant Peripheral Pulmonary Lesions. Front. Oncol..

[19] Wei H., Wang Y., Li J., Wang Y., Lu L., Sun J., Wang X. (2024). Diagnosis of benign and malignant peripheral lung lesions based on a feature model constructed by the random forest algorithm for grayscale and contrast-enhanced ultrasound. Front. Oncol..

[20] Tee A., Wong A., Yusuf G.T., Rao D., Sidhu P.S. (2020). Contrast-enhanced ultrasound (CEUS) of the lung reveals multiple areas of microthrombi in a COVID-19 patient. Intensive Care Med..

[21] Jacobsen N., Pietersen P.I., Nolsoe C., Konge L., Graumann O., Laursen C.B. (2020). Clinical Applications of Contrast-Enhanced Thoracic Ultrasound (CETUS) Compared to Standard Reference Tests: A Systematic Review. Ultraschall Med..

[22] Mathis G. (2017). Chest Sonography.

[23] Soldati G., Giannasi G., Smargiassi A., Inchingolo R., Demi L. (2020). Contrast-Enhanced Ultrasound in Patients with COVID-19: Pneumonia, Acute Respiratory Distress Syndrome, or Something Else?. J. Ultrasound Med..

[24] Rafailidis V., Andronikou S., Mentzel H.J., Piskunowicz M., Squires J.H., Barnewolt C.E. (2021). Contrast-enhanced ultrasound of pediatric lungs. Pediatr. Radiol..

[25] Rafailidis V., Huang D.Y., Yusuf G.T., Sidhu P.S. (2020). General principles and overview of vascular contrast-enhanced ultrasonography. Ultrasonography.

[26] Calliada F., Campani R., Bottinelli O., Bozzini A., Sommaruga M.G. (1998). Ultrasound contrast agents: Basic principles. Eur. J. Radiol..

[27] Ignee A., Atkinson N.S., Schuessler G., Dietrich C.F. (2016). Ultrasound contrast agents. Endosc. Ultrasound.

[28] Piscaglia F., Bolondi L. (2006). The safety of Sonovue in abdominal applications: Retrospective analysis of 23188 investigations. Ultrasound Med. Biol..

[29] Jang J.Y., Kim M.Y., Jeong S.W., Kim T.Y., Kim S.U., Lee S.H., Suk K.T., Park S.Y., Woo H.Y., Kim S.G. (2013). Current consensus and guidelines of contrast enhanced ultrasound for the characterization of focal liver lesions. Clin. Mol. Hepatol..

[30] Görg C., Bert T., Kring R., Dempfle A. (2006). Transcutaneous contrast enhanced sonography of the chest for evaluation of pleural based pulmonary lesions: Experience in 137 patients. Ultraschall Med..

[31] Linde H.N., Holland A., Greene B.H., Görg C. (2012). Contrast-enhancend sonography (CEUS) in pneumonia: Typical patterns and clinical value-a retrospective study on n = 50 patients. Ultraschall Med..

[32] Sidhu P., Cantisani V., Dietrich C., Gilja O., Saftoiu A., Bartels E., Bertolotto M., Calliada F., Clevert D.-A., Cosgrove D. (2018). The EFSUMB Guidelines and Recommendations for the Clinical Practice of Contrast-Enhanced Ultrasound (CEUS) in Non-Hepatic Applications: Update 2017 (Long Version). Ultraschall Der Med.-Eur. J. Ultrasound.

[33] Safai Zadeh E., Görg C., Prosch H., Jenssen C., Blaivas M., Laursen C.B., Jacobsen N., Dietrich C.F. (2022). WFUMB Technological Review: How to Perform Contrast-Enhanced Ultrasound of the Lung. Ultrasound Med. Biol..

[34] Caremani M., Benci A., Lapini L., Tacconi D., Caremani A., Ciccotosto C., Magnolfi A.L. (2008). Contrast enhanced ultrasonography (CEUS) in peripheral lung lesions: A study of 60 cases. J. Ultrasound.

[35] Schlingemann R.O., Rietveld F.J., de Waal R.M., Bradley N.J., Skene A.I., Davies A.J., Greaves M.F., Denekamp J., Ruiter D.J. (1990). Leukocyte antigen CD34 is expressed by a subset of cultured endothelial cells and on endothelial abluminal microprocesses in the tumor stroma. Lab. Investig..

[36] Fina L., Molgaard H.V., Robertson D., Bradley N.J., Monaghan P., Delia D., Sutherland D.R., Baker M.A., Greaves M.F. (1990). Expression of the CD34 gene in vascular endothelial cells. Blood.

[37] Siemerink M.J., Klaassen I., Vogels I.M., Griffioen A.W., Van Noorden C.J., Schlingemann R.O. (2012). CD34 marks angiogenic tip cells in human vascular endothelial cell cultures. Angiogenesis.

[38] Nowak-Sliwinska P., Alitalo K., Allen E., Anisimov A., Aplin A.C., Auerbach R., Augustin H.G., Bates D.O., van Beijnum J.R., Bender R.H.F. (2018). Consensus guidelines for the use and interpretation of angiogenesis assays. Angiogenesis.

[39] Safai Zadeh E., Keber C.U., Dietrich C.F., Westhoff C.C., Günter C., Beutel B., Alhyari A., Trenker C., Görg C. (2021). Perfusion Patterns of Peripheral Pulmonary Granulomatous Lesions Using Contrast-Enhanced Ultrasound (CEUS) and Their Correlation with Immunohistochemically Detected Vascularization Patterns. J. Ultrasound Med..

[40] Suresh K., Shimoda L.A. (2016). Lung Circulation. Compr. Physiol..

[41] Yuan A., Chang D.B., Yu C.J., Kuo S.H., Luh K.T., Yang P.C. (1994). Color Doppler sonography of benign and malignant pulmonary masses. AJR Am. J. Roentgenol..

[42] Hsu W.H., Chiang C.D., Chen C.Y., Kwan P.C., Hsu J.Y., Hsu C.P., Ho W.L. (1998). Color Doppler ultrasound pulsatile flow signals of thoracic lesions: Comparison of lung cancers and benign lesions. Ultrasound Med. Biol..

[43] Irodi A., Cherian R., Keshava S.N., James P. (2010). Dual arterial supply to normal lung: Within the sequestration spectrum. Br. J. Radiol..

[44] Eldridge L., Moldobaeva A., Zhong Q., Jenkins J., Snyder M., Brown R.H., Mitzner W., Wagner E.M. (2016). Bronchial Artery Angiogenesis Drives Lung Tumor Growth. Cancer Res..

[45] Eldridge L., Wagner E.M. (2019). Angiogenesis in the lung. J. Physiol..

[46] Sartori S. (2013). Contrast-enhanced ultrasonography in peripheral lung consolidations: What’s its actual role?. World J. Radiol..

[47] Safai Zadeh E., Westhoff C.C., Keber C.U., Trenker C., Dietrich C.F., Alhyari A., Mohr C.G.L., Görg C. (2021). Perfusion Patterns of Peripheral Organizing Pneumonia (POP) Using Contrast-Enhanced Ultrasound (CEUS) and Their Correlation with Immunohistochemically Detected Vascularization Patterns. Diagnostics.

[48] Yuan X., Zhang J., Ao G., Quan C., Tian Y., Li H. (2012). Lung cancer perfusion: Can we measure pulmonary and bronchial circulation simultaneously?. Eur. Radiol..

[49] West J.B., Luks A.M. (2020). West’s Respiratory Physiology: The Essentials.

[50] Suzuki J., Kojima M., Aokage K., Sakai T., Nakamura H., Ohara Y., Tane K., Miyoshi T., Sugano M., Fujii S. (2019). Clinicopathological characteristics associated with necrosis in pulmonary metastases from colorectal cancer. Virchows Arch..

[51] Nagy J.A., Chang S.H., Dvorak A.M., Dvorak H.F. (2009). Why are tumour blood vessels abnormal and why is it important to know?. Br. J. Cancer.

[52] Liu Y., Kan Y., Zhang J., Li N., Wang Y. (2021). Characteristics of contrast-enhanced ultrasound for diagnosis of solid clear cell renal cell carcinomas ≤ 4 cm: A meta-analysis. Cancer Med..

[53] Safai Zadeh E., Huber K.P., Görg C., Prosch H., Findeisen H. (2024). The Value of Contrast-Enhanced Ultrasound (CEUS) in the Evaluation of Central Lung Cancer with Obstructive Atelectasis. Diagnostics.

[54] Findeisen H., Trenker C., Figiel J., Greene B.H., Görg K., Görg C. (2019). Vascularization of Primary, Peripheral Lung Carcinoma in CEUS-A Retrospective Study (n = 89 Patients). Ultraschall Med..

[55] Trenker C., Wilhelm C., Neesse A., Rexin P., Görg C. (2018). Contrast-Enhanced Ultrasound in Pulmonary Lymphoma: A Small Pilot Study. J. Ultrasound Med..

